# Attachment Style and Internet Addiction: An Online Survey

**DOI:** 10.2196/jmir.6694

**Published:** 2017-05-17

**Authors:** Christiane Eichenberg, Markus Schott, Oliver Decker, Brigitte Sindelar

**Affiliations:** ^1^ Sigmund Freud PrivatUniversität Wien Vienna Austria; ^2^ Universtität Leipzig Leipzig Germany

**Keywords:** Internet, addictive behavior, surveys and questionnaires, Rorschach test

## Abstract

**Background:**

One of the clinically relevant problems of Internet use is the phenomenon of Internet addiction. Considering the fact that there is ample evidence for the relationship between attachment style and substance abuse, it stands to reason that attachment theory can also make an important contribution to the understanding of the pathogenesis of Internet addiction.

**Objective:**

The aim of this study was to examine people’s tendency toward pathological Internet usage in relation to their attachment style.

**Methods:**

An online survey was conducted. Sociodemographic data, attachment style (Bielefeld questionnaire partnership expectations), symptoms of Internet addiction (scale for online addiction for adults), used Web-based services, and online relationship motives (Cyber Relationship Motive Scale, CRMS-D) were assessed. In order to confirm the findings, a study using the Rorschach test was also conducted.

**Results:**

In total, 245 subjects were recruited. Participants with insecure attachment style showed a higher tendency to pathological Internet usage compared with securely attached participants. An ambivalent attachment style was particularly associated with pathological Internet usage. Escapist and social-compensatory motives played an important role for insecurely attached subjects. However, there were no significant effects with respect to Web-based services and apps used. Results of the analysis of the Rorschach protocol with 16 subjects corroborated these results. Users with pathological Internet use frequently showed signs of infantile relationship structures in the context of social groups. This refers to the results of the Web-based survey, in which interpersonal relationships were the result of an insecure attachment style.

**Conclusions:**

Pathological Internet use was a function of insecure attachment and limited interpersonal relationships.

## Introduction

### Background

Nowadays the Internet is a central aspect of everyday life. There are many opportunities for online dating, problems can be discussed in thematically appropriate forums, and doctors can be consulted [1,2]. Even university education is shifting toward the net. Due to the interactive options afforded, the Internet has firmly established itself within everyday life [3]. However, negative aspects of the Internet are increasingly brought to the attention of the public [4]. For instance, certain Web-based self-help forums on problematic areas such as suicidality [5], self-injurious behavior [6], cyberbullying [7], or negative effects on health-related Internet use [8], are controversially discussed both within the media and among professionals.

### Internet Addiction

One of the clinically relevant problems Internet use may yield is Internet addiction. Even though most people use the Internet every day without problems, recent figures highlight that excessive Internet use is a significant problem [9]. As a unified concept of the disorder and related diagnostic tools are not yet available [10], prevalence estimates demonstrate high levels of variance with values ranging from 1.5-11.6%. A review article found an overall prevalence of 3-5% for computer game and Internet addiction.

With regard to the many behaviors associated with the Internet, the question arises as to whether “Internet addiction” concerns a singular disorder, or whether the only commonality among various Internet-related behavior disorders is the use of the same medium (for the concept of specific Internet addiction, see Davis [11]). Accordingly, excessive Internet use can refer to different Web-based services, such as Web-based computer games [12], gambling [13], sexual content [14], or communicative apps such as chat or social networks [15] that differ in their “addictive” potential. Of particular concern for addiction behaviors are Web-based role-play games and sexual content [16].

Another question relating to the independence of the disorder “Internet addiction” has been discussed for many years [17]: Is “Internet addiction” an independent clinical disorder or rather a concomitant symptom of some other mental illness? Neurobiological research contributes to the answer by showing the equivalence of substance-related addictions and behavioral addictions [18]. This finding is mainly based on the neurobiological correlations between substance-related and behavioral addictions [19]. These results are part of a growing body of research that advocates Internet addiction as a behavioral addiction.

Behavioral addiction is a relatively new term for excessive behavior, which depicts characteristics of psychological dependence [20,21]. The term “behavioral addiction” marks the behavior of the user, and not the medium as the pathological object. In the latest edition of the Diagnostic and Statistical Manual of Mental Disorders, Fifth Edition (DSM-5), behavioral addictions have been explicitly included; the chapter previously named “Substance-Related Disorders” was renamed into “Addiction and Related Disorders.” The diagnostic criteria of a behavioral addiction are based on the criteria known for substance dependencies, that is, development of tolerance, withdrawal symptoms, unsuccessful attempts to reduce consumption, neglecting other areas, and use despite negative consequences [22]. Although Internet addiction does not constitute an autonomous clinical entity, Internet Gaming Disorder is listed in the appendix of DSM-5.

The inclusion in Section III of DSM-5 outlines the importance of Internet-related addictions, emphasizing the need for further research in this area. Thus, further studies investigating the etiology and pathogenesis are necessary. Furthermore, it is crucial to identify variables and dispositions that determine the etiology of Internet addiction in order to develop effective therapeutic measures.

### Attachment Theory

In line with integrative explanations for substance use disorders, complex etiopathogenic models are also employed for Internet addiction. For instance, Wölfling et al [23] developed an integrative model with an emphasis on learning theory and neurobiological mechanisms in the context of personality traits. Additionally, Internet addiction can be explained using cognitive-behavioral [24], psychodynamic approaches [25], as well as cultural and social considerations [26]. In this context, attachment theory can also make an important contribution to understanding preconditions associated with the development of behavior addictions, due to the widely documented relationship between attachment experiences and substance dependency [27]. Similar mechanisms underlying substance dependence are therefore both feasible and plausible for Internet addictions. For example, bypassing situations where patterns of attachment are activated by excessively spending time on the Internet or replacing negative relationship experiences by rewarding Web-based activity [28]. Overall, as a social medium and the relationship component contained therein, the Internet—compared with other addictive substances—provides even more possibilities to manage deficient attachment and relationship patterns. For example, online interaction in social networks, chats, and forums can dampen feelings of social isolation of people with an uncertain attachment style. In particular, the possibility of anonymous communication over the Internet plays a vital role in compensating social isolation via online contacts and relationships [29].

### Research Question

On the basis of the discussed findings, the aim of this study was to investigate the relationship between attachment style, motives for use, used services, and Internet addiction. It was hypothesized that, in comparison with securely attached users, significantly more insecurely attached users would display an Internet addiction. Furthermore, it was assumed that users with an insecure attachment style would differ in their motives for use compared with users with a secure attachment style. It was also hypothesized that users with an Internet addiction would report different motives for use compared with users without an Internet addiction. Finally, it was assumed that users with an insecure attachment style would use Web-based services more often than users with a secure attachment style.

## Methods

### Design

A Web-based survey was carried out. The Web-based questionnaire was distributed both on Facebook and on 15 thematically different forums (ranging from parent-, travel-, computer- to craft- and comic platforms) to obtain the most heterogeneous sample possible. The survey period lasted for 6 weeks. For descriptive inferential data analysis and hypothesis testing, SPSS version 20 (IBM, Somers, NY, USA) was used.

To rule out methodological artifacts in the first quantitative self-assessment study, a second qualitative study was conducted. In light of the limitations given in any self-evaluation study, the prior aim of this methodological triangulation study was to gain data that for sure could not be influenced by intentional or unintentional bias. Therapists in Austrian clinics as well as the University Clinic of Mainz were contacted to recruit possible participants for the Rorschach study.

### Sociodemographic Data

Age, gender, relationship status, and the duration of the existing partnership, highest level of education as well as current job situation were assessed. Furthermore, information about duration and frequency of Internet usage was collected.

#### Bielefelder Partnership Expectations Questionnaire

The Bielefelder partnership expectations questionnaire was used to assess the attachment style of participants. This inventory consists of 31 items that are rated on a 5-point Likert scale ranging from 0 (*completely disagree*) to 4 (*completely agree*).

The questionnaire evaluates the following five attachment styles: *secure*, *conditionally secure*, *avoidant-closed*, *ambivalent-clingy*, and *ambivalent-closed*. The reliability of the scales (Cronbach alpha=.77 to .89) is satisfactory.

The Bielefeld questionnaire is different from others in two ways: (1) attachment style is operationalized as configurations of scale scores, which allow qualitative distinctions in terms of functioning and (2) five empirically identified attachment styles are distinguished. Nonetheless validation of the classifications with a German translation of the “Adult Attachment Scale (AAS)” yielded good results [30].

#### Online Addiction Scale

The online addiction scale is a diagnostic tool consisting of 14 items about Internet addiction that are rated on a 5-point Likert scale with a maximum possible total score of 27 points. Cutoff values differentiate three different user types: *normal* (<7 points), *problematic* (>7 points and <13 points), and *pathological* (>13 points).

In addition, the questionnaire assesses how frequently participants used the following 8 different Web-based services: Web-based games, shopping, chatting in forums, writing emails, Web-based sex services, Web-based gambling, Web-based communities, and information retrieval. The frequency of use is reported on a 4-point scale ranging from 0 (*never*) to 3 (*very often*).

Reliability, validity, and utility of the instrument have been confirmed showing a good internal consistency of .88 and homogeneity of .34. An exploratory factor analysis yielded a one-way solution confirming factorial validity [31].

#### Cyber Relationship Motive Scale (CRMS-D)

The Cyber Relationship Motive Scale is a self-evaluation of user’s relationship motives for going online. Survey respondents are prompted to rate how well each of the 27 possible items applied to their motives for Internet usage on a 5-point scale ranging from 0 (*strongly agree*) to 4 (*strongly disagree*): anonymity, opportunity to meet new people, simple communication, curiosity, emotional support, social contact, escape from the real-world, finding love or a sexual partner.

A confirmatory factor analysis was conducted yielding a goodness-of-fit index of .90. On the basis of the factor loadings, acceptable validity could be determined [32].

#### Rorschach Inkblot Test

The Rorschach Inkblot Test is a performance-based personality test. The test consists of 10 inkblot stimuli: 5 are achromatic and 5 include chromatic colors. Examinees look at each inkblot and say what it looks like or what it might be. Examinees can give one or more responses per inkblot. Following test administration, Rorschach responses are coded and tallied to form main variables, such as “Situational Stress,” “Affective Features,” “Interpersonal Perception,” or “Self-Perception.” The interpretation for each Rorschach variable is guided by interpretive paragraphs that are sequentially arranged in the test manual. The Rorschach variables are given a cutoff score that indicates which interpretive paragraph to choose. To determine the degree to which the results statistically deviate from the norm, the examiner must compare each of the variables with the relevant descriptive statistics that are reported in large normative tables [33].

The Rorschach test can be described as “immune” to any form of bias. This immunity is due to the fact that the evaluation of the Rorschach test is not carried out by an interpretation of the subject’s responses; instead responses are coded by established criteria. Although those established criteria differ based on the evaluation system applied, they generally follow the same basics: acquisition, the experience, and content [34]. This complex evaluation strategy is by no means transparent for subjects; as such any attempt at influencing the interpretation is impossible. This criterion is impossible to be met by any questionnaire or any narrative projective test (like Thematic Apperception Test) but only by a performance-based test analyzing the performances of the client quantitatively [34,35].

As the Rorschach Inkblot Method (RIM) had provoked numerous discussions about its reliability, validity, and utility, it has received a maybe more intensive level of scrutiny than any other personality test, summarized in a meta-analysis and reproved in an independent blue ribbon panel. Taken together, studies on reliability and validity of the RIM showed the same or even more valid results for the RIM as for other well-validated inventories [36,37].

## Results

### Sample

During the survey period, the Web-based questionnaire was accessed N=1009 times. Around 39,74% (401/1009) of participants did not proceed past the start page, another 28.35% (286/1009) had dropped out by page 3. However, as only a further few participants dropped out at later stages of the survey, the dropout rate can be deemed acceptable [38].

Overall, the questionnaire was completed 249 times, constituting 24.86% of total page views. After checking for plausibility of answers, 4 records were removed, resulting in a total sample of 245 participants (168 female, 77 male) aged 16-61 years (mean 29.6, SD 9.17).

At the time of the survey, 78.8% (193/245) were in a partnership, with an average relationship duration of mean 77.2 months (SD 101.21). Less than half (40.81%) of subjects (100/245) were employed full-time or part-time, one-quarter of the sample consisted of students (62/245, 25.31%), a further 5.71% (14/245) were also working in addition to studying, a small fraction of the total sample were unemployed (16/245, 6.53%), few participants (16/245, 6.53%) were self-employed, and 2.86% were trainees (7/245). In total, 30 subjects (12.2%) reported “other” in terms of employment.

The Rorschach Inkblot Test was conducted with a small sample of 16 voluntary male subjects. Although 8 participants showed abusive Internet usage and 3 of them met the criteria for Internet addiction, a control group of 8 subjects with nonabusive Internet usage was recruited. Participants were aged between 18 and 47 years (mean 31 years). Subjects were primarily students, full-time employees, or self-employed.

### Duration and Frequency of Internet Use

On average, study participants had used the Internet for over 10 years (mean 10.91, SD 3.92). The daily use of the Internet for private purpose amounted to an average of mean 4.35 h (SD 4.27), ranging from a minimum of 0.1 h up to 21 h a day. A majority of the sample (93.1%, 228/245) used the Internet daily, only 6.9% (17/245) went online 2-3 times per week. None of the subjects stated that they used the Internet once a week, once a month, or less.

#### Online Services

Subjects used the Internet mainly for online shopping (mean 2.32, SD 0.72). Other popular services included online research (mean 2.32, SD 0.72) and social platforms (mean 2.02, SD 1.11), emails (mean 2.02, SD 0.85), as well as chats and forums (mean 1.55, SD 1.06). In contrast, games (mean 0.70, SD 0.99), sexual content (mean 0.51, SD 0.79), and gambling (mean 0.12, SD 12.43) were less popular.

#### Online Relationship Motives

Simple communication (mean 3.47, SD 1.02) and the opportunity to find new friends (mean 3.20, SD 0.95) were the dominant motives for Internet use. Emotional support (mean 2.37, SD 0.94), anonymity (mean 2.04, SD 1.3), and escapism (mean 2.03, SD 1.17) appear to be motives of medium importance. The lowest, sexual (mean 1.59, SD 1.07), and social-compensatory motives (mean 1.69, SD 1.14), were less frequently reported as online relationship motives.

#### Attachment Style

More than half (50.6%, 124/245) of participants showed an insecure attachment style (*ambivalent-closed*, *ambivalent-clingy*, and *avoidant-closed*), whereas almost as many subjects showed a secure attachment style (*conditionally secure* and *secure*). Therefore, secure and insecure attachment style was equally represented in the sample (Table 1).

**Table 1:** Frequency distribution of the variable attachment style.

Attachment Style			Frequency, n (%)
**Secure attachment**			121 (49.4)
	Secure	25 (10.2)	
	Conditionally secure	96 (39.2)	
**Insecure attachment**			124 (50.6)
	Avoidant-closed	50 (20.4)	
	Ambivalent-clingy	36 (14.7)	
	Ambivalent-closed	38 (15.5)	
Overall			245 (100)

#### Internet Addiction

Only 1.2% of subjects (3/245) could be classified as addicted Internet users. To allow for appropriate statistical analysis, the categories “pathological” and “problematic use” were merged to the variable *Internet addiction tendency* with 30 subjects. A majority (87.3%, 214/245) of users showed normal Internet use.

#### Attachment Style and Internet Addiction

It was hypothesized that, in comparison with securely attached users, significantly more insecurely attached users would display an Internet addiction. Descriptive data revealed that the majority of participants with a tendency for Internet addiction (n=24) were categorized as insecurely attached, and only a few (n=6) were securely attached. An opposite trend can be detected when considering subjects without Internet addiction tendencies. More subjects categorized as securely attached (n=115) showed no tendencies for an Internet addiction than insecurely attached subjects (n=99).

A chi-square test was conducted. Unsurprisingly, it was found that insecurely attached subjects differ significantly from securely attached subjects in their tendency for Internet addiction (χ²_1_=12.0, *P*=.003). Furthermore, differences regarding Internet addiction tendencies between the five attachment categories were significant (χ²_4_=27.09, *P*=.004). Most strikingly, an ambivalent attachment style was associated with Internet addiction. More than two-third (n=21) of all subjects, who showed Internet addiction tendencies (n=30), could be allocated to this category, although occupation of this category was generally rather low (n=52).

#### Attachment Style and Motives for Use

A univariate test of between-subjects effects (analysis of variance, ANOVA) was conducted to investigate the relationships between attachment style and motives for Internet use. A significant result was found for two motives, that is, *anonymity* (*F*_4_=2.82, *P*=.02) and *emotional support* (*F*_4_=3.16, *P*=.03). For subjects with an insecure attachment, the motives “emotional support” (mean 2.66) and “anonymity” (mean 2.36) were significantly more important than for subjects with secure attachment (emotional support, mean 2.03; anonymity, mean 1.67). To check for significant differences between the three attachment styles, post hoc single comparisons were performed. Bonferroni correction was used to adjust the significance threshold for multiple comparisons. There were significant differences between users with the attachment style *ambivalent-closed* and *conditionally secure* (P *<*.001) and *secure* (*P*=.02) with respect to the motives anonymity and *emotional support*. Ambivalent-closed participants showed significantly higher values in the motives *anonymity* (mean 2.84) and *emotional support* (mean 2.56) than *conditionally secure* (anonymity, mean 2.10; emotional support, mean 1.68) or *secure* users (anonymity, mean 1.79; emotional support, mean 1.63).

#### Internet Addiction and Online Relationship Motives

It was checked whether people with Internet addiction tendencies differed significantly in their online relationship motives compared with normal Internet users. A comparison of means revealed significant differences in the online relationship motives *anonymity* (*t*_159_=−4.42, *P*=.003), *simplified communication* (*t*_39.72_=−3.38, *P*=.006), *emotional support* (*t*_159_=−3.74, *P*<.001), *social compensation* (*t*_27.72_=−2.13, *P*=.04), and *escapism* (*t*_159_=−4.88, *P*<.001).

**Figure 1:**
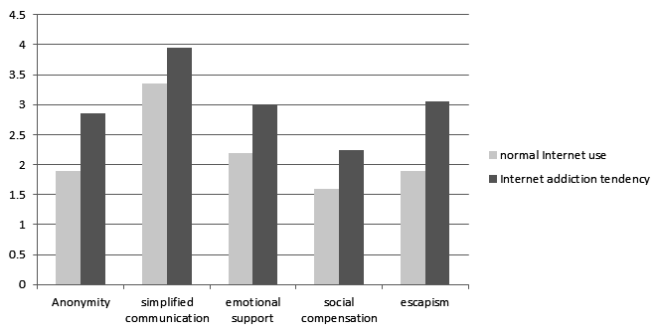
Internet addiction and online relationship motives.

For all motives, participants with Internet addiction tendencies had higher mean values (see Figure 1).

#### Attachment Style and Internet Services

A multivariate analysis of variance (MANOVA) was performed to examine the relationship between attachment style and Web-based services. Using the conservative Pillai trace, no significant main effect for the factor attachment style could be found. Thus, the hypothesis that the use of various Web-based services and apps is associated with different binding styles can be rejected.

#### Internet Addiction Tendencies and Internet Services

There were significant differences between groups with and without Internet addiction tendencies with regard to the use of Web-based sex services (*t*_32.219_=−3.20, *P*=.002, *d*=0.84) and the use of chats and forums (*t*_242_=−2.09, *P*=.04, *d*=0.40). The comparison of means showed that both services were more frequently used by subjects with Internet addiction tendencies (forums or chats: mean 2.93, SD 1.01; Web-based sex services: mean 2.1, SD 1.12) than by modest users (forums or chats: mean 2.5, SD 1.06; Web-based sex services: mean 1.43, SD 0.70).

#### Rorschach Inkblot Test

The analysis of the Rorschach protocols gave the following results: patients who reported a tendency to pathological Internet use showed a significantly lower degree of sociability (as measured by H% [percentage of human figures], *P*=.05, Mann-Whitney *U* test), whereas a higher level of accuracy of perception (measured on F+% [form level], *P*=.05, Mann-Whitney *U* test) occurred in the control group of patients who denied a tendency to pathological Internet use. Examination of the homogeneity of variance using Levene test proved the parameters of the percentage of intermediate figure responses (“S-responses”) that are seen as an indicator for the potential of aggression, as significant (*P*<.001). In the group of abusive patients, the minimum percentage of S-responses was at 0% and the maximum at 38.6%, whereas in the group of inconspicuous Internet users, the minimum was at 10.64% and the maximum was at 25%. Therefore, the group of abusive Internet users can be regarded not only as particularly aggression-inhibited, but also as aggressive patients. Of particular interest in this context is the common assumption that the online world can both dissipate and fuel aggression.

## Discussion

### Principal Findings

Although a growing body of scientific literature highlights that Internet addiction is a serious health problem, there have been no etiopathological studies to support this claim. Since an association between secure or insecure attachment and substance dependence is well-documented, the aim of this study was to examine whether people differ in their Internet addiction tendencies in regards to their attachment style.

The assumption that insecure attached people show higher Internet addiction tendencies could be confirmed. Ambivalent attachment styles were particularly associated with pathological Internet usage tendencies. For ambivalent-closed attached people, the motives *anonymity* and *social support* were significantly more important than for secure and conditionally secure users. Furthermore, participants with Internet addiction tendencies usage identified anonymity, emotional support, escapism, and social compensation as important relationship motives for Internet use.

However, attachment styles were not associated with the Web-based services used. Nonetheless, there was a difference in the use of Web-based services with regards to the Internet addiction tendencies. In particular, participants with Internet addiction tendencies engaged significantly more in Web-based chats and forums than normal users. Thus, the possibility of Web-based communication seems to play a prevalent role in the context of pathological Internet use.

It appears therefore that, in comparison with Web-based services used, motivational factors relating to online relationships can best explain this finding. In contrast, the relationship between the Web-based services used and the tendency toward Internet addiction can be attributed to the individual addictive potential of the particular Web-based service in question.

Overall, the findings of the Rorschach Inkblot Test are in line with the results of the Web-based survey, in which abusive Internet use presents itself as a function of insecure attachment and impaired interpersonal relationships, that is, the preference for chats and forums by study participants with tendencies toward Internet addiction as a result of an infantile relationship that has not reached the level of group compatibility. Chats and forums offer fictional contact with a group, while communication between users remains in virtual space, which in turn significantly weakens the experience of group presence. Thus, communication in chat-rooms or forums is within the framework of a group constellation, however, always experienced at the level of primary intersubjectivity, that is, within the relationship between I and You. Considering interpersonal skills as an expression of attachment capability [39], it is striking that the intersubjectivity of abusive Internet users has not reached the level of maturity of secondary intersubjectivity [40,41].

These results illustrate the escapist and social-compensatory function associated with pathological Internet usage. Although ambivalent-closed users demonstrate difficulties with acceptance and opening up to others, a parallel desire to connect with others also exists [42]. The media-ecological framework model according to Döring [3] and the uses and gratifications [43] approach can help to understand these findings. On this basis, it can be assumed that the Internet offers specific opportunities for interaction that people with a high Internet addiction tendency do perceive as sufficient forms of communication. Consequently, they report Web-based communication to be more easily accessible and understandable. Apparently, these people appear to be able to use the medium to their advantage, they seem to be able to build a relationship world online in which they can experience emotional support and balance social restrictions. At the same time, the network offers the possibility to temporarily remove a burden from a distressed reality. The factor “anonymity” therefore takes a position of particular importance. The anonymity of the Internet allows for a new presentation of the self, whereby ambivalent-closed users are able to compensate for fears associated with acceptance. At the same time, however, the anonymity provides a platform for online disinhibition [44], which may increase the willingness to open up to others. It appears that it is predominantly users with an ambivalent-closed style attachment who use the Internet in order to compensate for “real” deficits, demonstrating therefore the clearest trend toward pathological Internet use [45]. The importance of relationship motives for this group supports the hypothesis that a socially compensatory component is key for a high Internet addiction tendency.

The satisfaction of these motives is in line with the needs relevant in the context of media usage postulated in Schramm and Hasebrink [46]. Above all, social needs, as well as the search for relaxation and escapism can be satisfied online. At the same time, the fulfillment of an existing need for social affection in the context of an otherwise unfavorable binding pattern plays a particular important role. At this point, it becomes clear to what extent the cognitive-behavioral model of the Internet addiction is appropriate in this context. Although on the one hand side attachment styles [47] can have an unfavorable influence on the self-concept, on the other side online social support and social compensation can act as a reinforcing stimulus. This can result in cognitions suggested by Davis [11], which view the self and others only online pleasantly. Strengthening of this experience might result in an abusive and even addictive Internet usage behavior.

Overall, this study allows for important conclusions about the background of pathological Internet usage to be made. As a consequence, the presented findings are indicatory for future research regarding the etiopathology of Internet addiction.

### Limitations

Methodological issues limit the validity of the research results. For example, the lack of participants classified as “addicted” marks a key problem of this study. Therefore, conclusions can only be drawn for problematic, subclinical Internet use. In light of the generally low prevalence of the disorder of only 3-5% [9], it is recommended to conduct another study with a specially selected sample. It is also important to point out that users of Web-based games, gambling, and sex services were not adequately represented in the sample. This one-sided sample composition can be explained by solely recruiting participants on forums and social networks. Moreover, it would be important to find out to what extent abusive and pathological Internet use differs (ie, regarding comorbidity, used Web-based services and further disease progression). A long-term monitoring of users with Internet addiction tendencies would be required to reveal whether attachment style can act as a disposition and consequently facilitate the development of an Internet addiction. As such, adequate preventive measures could then be applied at an early stage.

In all Web-based surveys, the sample composition constitutes another issue limiting the value of the found results [48]. It may be that Internet users, who are striving to relativize the negative image of Internet dependency, predominantly responded to the survey. A related concern is the limited possibilities to gain information in a self-assessment process typical for Web-based studies. However, it should be noted that while the statistical evaluation can be regarded as objective, the analysis of values and their interpretation cannot be assumed for the data itself [49], due to the fact that every form of self-evaluation has an intentional or unintentional bias. This can include self-deception, simulation or dissimulation, or indeed socially desirable answers [41].

For the Rorschach test, it has to be taken into account that projective techniques are differing from structured tests in stimulus and response and therefore remain “problematic instruments from a psychometric standpoint” [50]. In projective techniques, the stimuli used are more ambiguous than in structured tests. Although items of structured personality tests based on self-observation also bear a certain degree of ambiguity, for example, by using the term “often,” that can be interpreted in various ways, projective techniques, in general, are providing a much wider freedom of response and consecutively provoking a much wider response variety in nature and number going together with a complex procedure of scoring. Therefore, they are much more vulnerable by interpreter´s scope of accuracy.

### Implications for Therapy

A tendency to pathological Internet use was associated with an insecure attachment style and limited interpersonal relationships; it therefore seems plausible to take therapeutic measures to help patients address real-life deficits, that is, this can be achieved by a therapeutic relationship with the therapist as a “substitute attachment figure” [28], or in a group therapy, where the therapeutic community can also provide corrective relationship experiences [51], and finally the results of the Rorschach test highlight the need for secondary and tertiary intersubjectivity in patients [52].

## Abbreviations

AAS: Adult Attachment Scale
ANOVA: analysis of variance
CRMS-D: Cyber Relationship Motive Scale
DSM-5: Diagnostic and Statistical Manual of Mental Disorders, Fifth Edition
MANOVA: multivariate analysis of variance
RIM: Rorschach Inkblot Method
